# Rapid palliation of symptoms with platinum-based chemotherapy plus cetuximab in recurrent oral cancer: a case report

**DOI:** 10.1186/1758-3284-2-3

**Published:** 2010-01-27

**Authors:** Ricard Mesía, Ramón Palmero, Mònica Cos, Esther Vilajosana, Silvia Vázquez

**Affiliations:** 1 Medical Oncology Department, Institut Català d'Oncologia (ICO), Hospitalet de Llobregat, Barcelona, Spain; 2 Radiology Department, Institut de Diagnòstic per la Imatge, Hospital de Bellvitge. Hospitalet de Llobregat, Barcelona, Spain

## Abstract

**Background:**

Symptom control is an important consideration in the choice of treatment for patients with recurrent and/or metastatic squamous cell carcinoma of the head and neck (SCCHN). Patients who demonstrate objective tumour responses to platinum-based chemotherapy are more likely to have symptom relief than those who do not have such responses. A phase III trial (EXTREME) showed that adding the epidermal growth factor receptor (EGFR)-targeting IgG1 monoclonal antibody cetuximab to first-line platinum-based chemotherapy significantly prolongs progression-free and overall survival and increases response rate compared with platinum-based chemotherapy alone. We report here the case of a 60-year old female with recurrent squamous cell carcinoma of the gum who had rapid palliation of symptoms and reduction of facial disease mass following treatment with a combination of carboplatin/5-fluorouracil (5-FU) and cetuximab.

**Case presentation:**

The patient was diagnosed with T4N0 M0 disease of the oral cavity in November 2006 and underwent surgery, with R0 resection, followed by adjuvant radiotherapy and concomitant cisplatin chemotherapy. Around 3 months later, the disease recurred and the patient had severe pain (9/10 on a visual pain scale), marked facial oedema and a palpable facial mass of 89 mm. The patient received 4 21-day cycles of carboplatin (AUC 5), 5-FU (1,000 mg/m^2^/day for 4 days) and cetuximab (400 mg/m^2 ^initial dose followed by subsequently weekly doses of 250 mg/m^2^), with continuation of cetuximab monotherapy at the end of this time, and pain relief with topical fentanyl and oral morphine. After 7 days of treatment, pain had reduced to 2/10, with discontinuation of morphine after 4 days, and the facial mass had reduced to 70 mm. After 2 cycles of treatment, the facial mass had decreased to 40 mm. After 3 cycles of treatment, pain and facial oedema had resolved completely and a cervical computed tomography scan showed a marked reduction in tumour mass. Cetuximab monotherapy was continued uninterrupted for 7 months.

**Conclusion:**

This case illustrates the rapid reduction of tumour mass and disease-associated pain and oedema that can be achieved with a combination of platinum-based chemotherapy and cetuximab in recurrent and/or metastatic SCCHN.

## Background

Cetuximab (Erbitux^®^) is an IgG1 epidermal growth factor receptor (EGFR)-targeting monoclonal antibody which has been shown in phase III trials to improve the survival of patients with squamous cell carcinoma of the head and neck (SCCHN) when combined with first-line platinum-based chemotherapy in recurrent and metastatic disease [1] and when combined with radiotherapy for the treatment of locoregionally advanced disease [2,3]. In the phase III EXTREME trial in recurrent and metastatic SCCHN, first-line treatment with a combination of platinum (cisplatin or carboplatin), 5-fluorouracil (5-FU) and cetuximab not only significantly prolonged progression-free and overall survival, compared with platinum-based chemotherapy alone, but also increased the overall tumour response rate by 16 percentage points (36% with cetuximab/platinum/5-FU vs 20% with platinum/5-FU alone) [1]. The tolerability of the two regimens was similar, with a few exceptions. As anticipated, grade 3/4 skin reactions and infusion-related reactions were confined to the cetuximab plus platinum-based chemotherapy arm of the trial. In addition, there were significantly more cases of grade 3/4 sepsis and hypomagnesaemia in the cetuximab/platinum/5-FU arm, compared with platinum-based chemotherapy alone, although the absolute numbers of patients were small. The effect of treatment on tumour response in this trial suggests that the combination of cetuximab and platinum-based chemotherapy may be particularly useful for symptomatic relief. We present here the case report of a patient with recurrent squamous cell carcinoma of the gum who derived rapid palliation of symptoms following treatment with a combination of cetuximab, carboplatin and 5-FU.

## Case presentation

### Initial diagnosis

In August 2006, a 60-year old Caucasian female (date of birth 02 February 1946) presented with a left paramandibular mass. Following a physical examination, cervical computed tomography (CT) scan and biopsy, a diagnosis of squamous cell carcinoma of the gum was made (November 2006). The patient had type II diabetes but had no history of smoking or regular alcohol consumption. On 11 December 2006, the patient underwent surgery comprising left mandibulectomy and left lip commissurotomy, with facial dermis resection, pectoral flap reconstruction and left supraomohyoid lymphadenectomy, and a tracheotomy. Pathological examination revealed grade 1 squamous cell carcinoma, 4.5 cm diameter by 4.0 cm deep, with skin extension throughout the mandibular border and involvement of the jaw bone. There was no lymph node involvement and no metastases were detected. Disease was staged as pT4N0 M0 following a thoracic X-ray. Tumour resection margins were free of disease. From 07 February 2007 to 26 March 2007, the patient received adjuvant radiotherapy (66 Gy) plus 3 doses of concomitant cisplatin (100 mg/m^2^, days 1, 22 and 43).

### Disease recurrence

**Figure 1:**
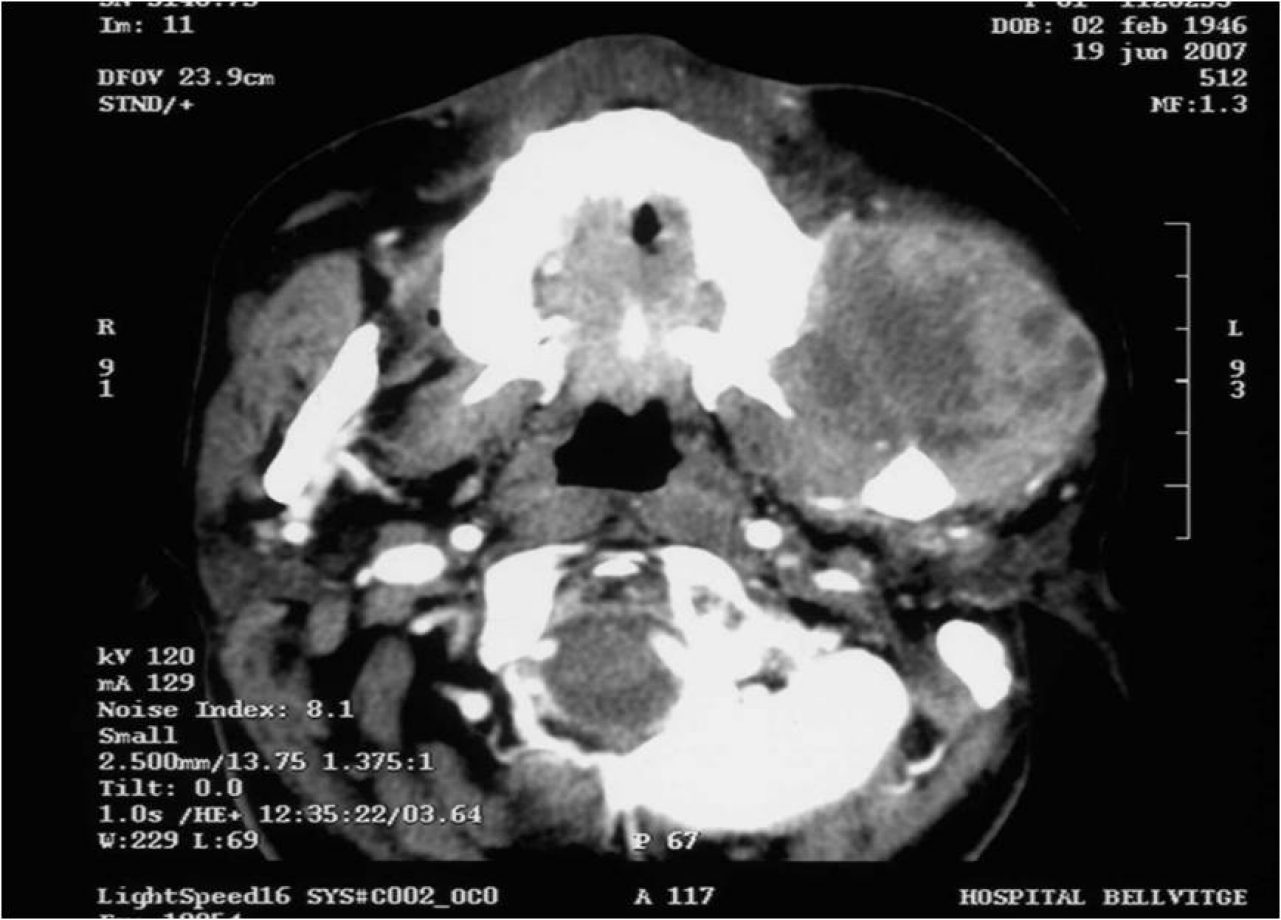
**Pre-treatment axial cervical contrast-enhanced computed tomography scan**. The scan shows a bulky, expansive, soft-tissue mass at the masticator space with heterogeneous enhancing and central necrosis. The infiltration of the masseter and medial pterigoid muscles is seen.

Two and a half months later (08 June 2007), the patient presented at the emergency unit with intense pain in her left hemiface. The patient was prescribed pain management with diclofenac (100 mg/12 hours, by suppository), gabapentin (300 mg/8 hours, orally) and fentanyl (25 μg/72 hours, topical administration), supplemented with additional doses of morphine as required (5 mg, orally). On the 18 June 2007, the patient made an initial visit to our clinic. The patient's pain had been controlled but she now reported symptoms of left periorbital parasthesia and grade 1 asthenia. On physical examination, the patient was assessed as having a Karnofsky performance status of 80%. Left hemifacial, lip, periorbital and cervical oedema were recorded. A cervical, contrast-enhanced CT scan on 19 June 2007 revealed a bulky, expansive soft-tissue mass, with central necrosis, extending from the left of the suprazygomatic masticator space to the masseter muscle insertion site. The temporalis, masseter and medial pterigoid muscles were infiltrated. The lesion was close to the maxilla and mandibular ramus. Cortical bone erosion was excluded. These findings were compatible with recurrent disease (Figure 1).

### Treatment regimen for recurrent disease

After assessing the extent of the disease recurrence and the structures involved, a multidisciplinary committee concluded that local treatment, such as salvage surgery or re-irradiation, would not be possible. Palliative treatment with chemotherapy was considered the best option and the patient agreed to this management approach. The patient was scheduled to receive treatment according to the regimen used in the investigational arm of the EXTREME trial. The choice of platinum agent was based on the fact that the patient had previously received cisplatin in combination with radiotherapy for locally advanced disease and that her glomerular filtration rate was 59 mL/min. The patient received 4 21-day cycles of treatment with carboplatin (AUC 5), 5-FU (1,000 mg/m^2^/day, continuous infusion for 4 days) and cetuximab (initial dose 400 mg/m^2^, with subsequent weekly doses of 250 mg/m^2^). Weekly cetuximab maintenance therapy was continued following the end of the 4 cycles. Analgesia comprised topical fentanyl (50 μg/72 hours) and oral morphine (2.5 mg/4 hours) as required.

### Response to treatment

Immediately prior to treatment (09 July 2007), the patient's condition had deteriorated since her first visit to the clinic and she reported severe pain, registering 9/10 on a visual pain scale, which was not controlled with analgesia. She had difficulty chewing and required her food to be mashed. Other symptoms included grade 2 asthenia and grade 1 anorexia. Physical examination revealed a reduction in the patient's Karnofsky performance status (to 60%), an increase in facial oedema and the presence of a palpable facial mass (89 mm diameter).

**Figure 2:**
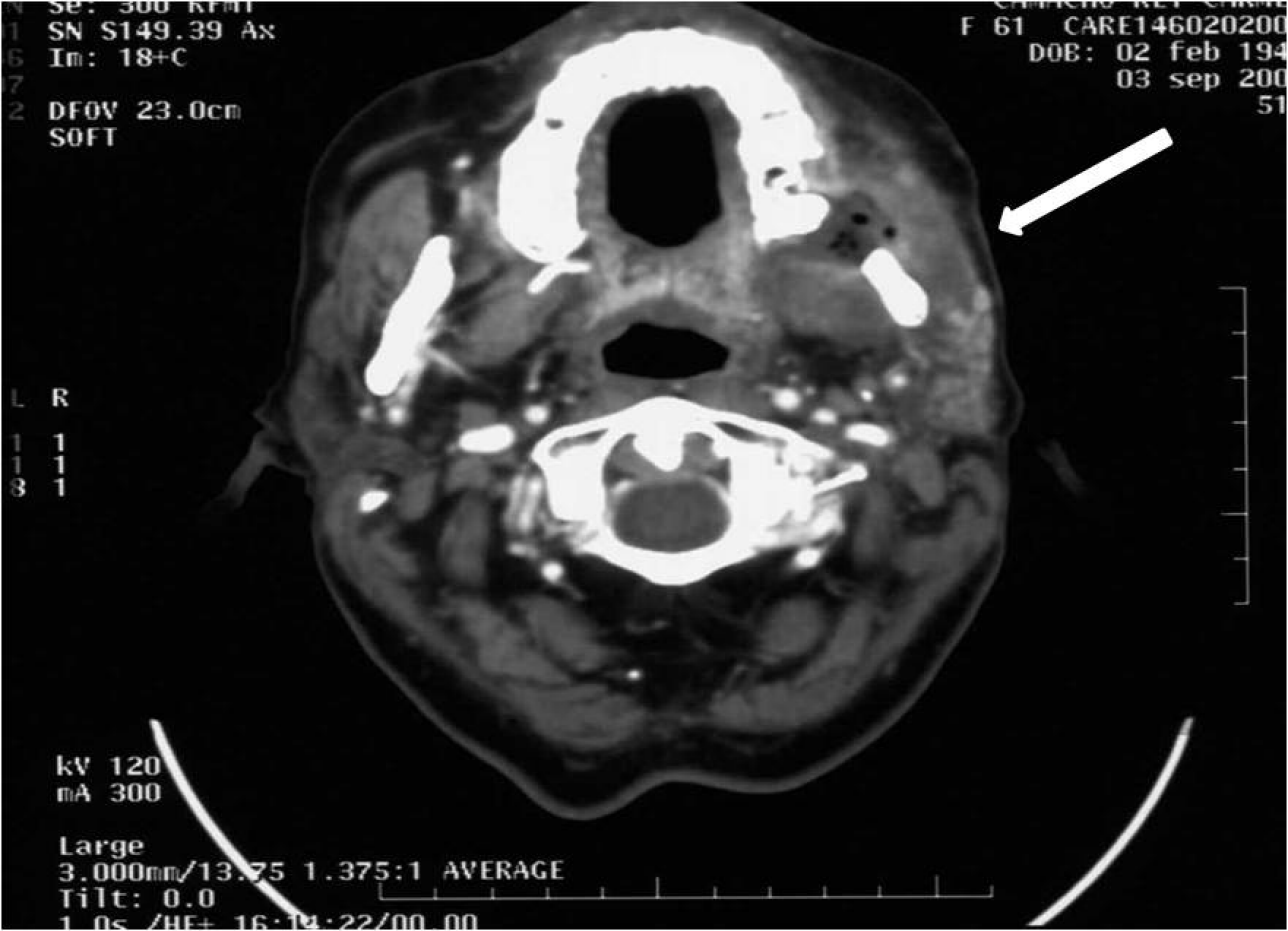
**Axial contrast-enhanced cervical computed tomography scan after 3 cycles of cetuximab plus platinum-based chemotherapy**. The scan shows a marked reduction in the tumor. A large hypodense area with air bubbles and peripheral enhancing corresponding to necrosis is seen in the masticator space. A partial tumor remission was considered.

After 4 days of treatment, the patient reported a reduction in pain and the use of oral morphine was discontinued (Table 1). After 7 days of treatment, the patient reported the pain to be controlled (2/10 on the visual scale) with topical fentanyl (50 μg/72 hours) and diclofenac (100 mg/12 hours, by suppository), and there was a reduction in the facial mass to 70 mm in diameter. After 2 cycles of treatment, labial and periorbital oedema were reduced and there was a further decrease in the facial mass to 40 mm in diameter. After 3 cycles of treatment, the patient reported no pain (0/10 on the visual scale), leading to discontinuation of fentanyl, and the facial oedema had resolved. The patient also reported an improvement in the ability to chew food. A cervical, contrast-enhanced CT scan at this stage (03 September 2007) revealed a marked reduction of the solid lesion. A large area of necrosis, with peripheral enhancing, was observed at the masticator space (Figure 2). A partial tumor remission was considered.

**Table 1:** Efficacy and toxicity of treatment

Treatment cycle	Effect of treatment on signs and symptoms	Adverse events
Pre-treatment	Pain 9/10* Facial mass 89 mm	N/A
1	Pain reduction to 2/10* after 7 days, with discontinuation of oral morphine after 4 days Reduction of facial mass to 70 mm	Grade 2 nausea
2	Labial and periorbital facial oedema reduced Reduction of facial mass to 40 mm	Grade 2 nausea Grade 2 neutropenia Next treatment cycle delayed by one week
3	Pain resolved (0/10)* and pain relief discontinued Facial oedema resolved CT scan: partial tumour remission, with marked reduction of retromandibular mass	Grade 2 anaemia
4	No pain relief requirement	Grade 2 anorexia Grade 2 vomiting Grade 2 asthenia Grade 2 mucositis Grade 3 anaemia (requiring red cell transfusion) Grade 1 acne-like rash (facial)
Cetuximab maintenance (7 continuous months of treatment)	No pain relief requirement	Grade 2 mucositis Grade 2 acne-like rash (painful lesions on the scalp) Cetuximab discontinued for 3 weeks

*Pain was assessed using a visual pain scale with a range from 0 to 10: the higher the score, the more severe the pain. CT = computed tomography.

Cetuximab maintenance therapy was continued uninterrupted for 7 months. At this time, it was stopped for 3 weeks following the development of NCI-CTCAE (National Cancer Institute-Common Terminology Criteria for Adverse Events, Version 3.0) grade 2 mucositis and grade 2 acne-like rash on the patient's scalp.

On the 17 March 2008, the mucositis had resolved and the skin toxicity had reduced to grade 1. However, there was a return of hemifacial pain (4/10 on the pain visual scale), for which the patient received topical fentanyl (25 μg/72 hours). Following reintroduction of cetuximab monotherapy, there was a reduction in pain (2/10 on the visual scale). Cetuximab-associated skin reactions on the scalp were controlled by topical treatment, according to the protocol at our centre. Grade 1 reactions were managed by the use of hydrating cream, topical corticosteroid cream (0.5% betamethasone, twice a day) and/or topical antibiotic cream for acne-like rash, with oral antihistamines to control itch. For grade 2 reactions, topical corticosteroid cream (0.5% betamethasone, twice a day) was used, with the topical antibiotic cream mupirocin (twice a day) for ulceration and infection, and oral antihistamines to control itch.

On 27 May 2008 cetuximab was discontinued following a CT scan showing signs of tumour progression. Pain had been controlled up to this point on topical fentanyl (25 μg/72 hours). The patient died from disease progression on 06 June 2008.

## Conclusions

More than 50% of newly diagnosed patients with head and neck cancer are not cured with standard treatment approaches and will suffer local (more than 80%) and/or distant (less than 15%) relapse despite appropriate, aggressive therapy [4]. For selected patients with a single, non-bulky recurrence, salvage surgery can be considered, although this is likely to result in some degree of functional deficit. An alternative option is re-irradiation, with or without concurrent chemotherapy, which may be beneficial in terms of locoregional control and survival, but which is associated with substantial toxicity. Indeed, in general, the use of re-irradiation should be limited to the setting of a clinical trial [5]. When local treatment is not an appropriate option, single-agent or combination chemotherapy, is the standard treatment choice for good performance status patients. For the majority of patients with a poor performance status (Eastern Cooperative Oncology Group [ECOG] 2 or worse, equivalent to Karnofsky performance status 60% or less [6]), like the patient discussed here, the only real option is best supportive care.

In patients receiving chemotherapy, the most commonly used schedule is one based on cisplatin combinations. For many years investigators have attempted to identify the gold standard first-line treatment, and randomised trials have compared the effects of cisplatin-based chemotherapy with other standard approaches or other cisplatin-based combinations [7,8]. In terms of response rate, combination chemotherapy was superior to monotherapy, but did not improve survival. In addition, cisplatin-based combination chemotherapy was associated with more toxicity than monotherapy. Thus, for 30 years, in recurrent head and neck cancer, no first-line combination chemotherapy regimen had demonstrated a survival advantage over platinum-based chemotherapy. Therefore, the best option for these patients was to be treated within the context of a clinical trial utilizing therapy containing molecules against new targets, such as cetuximab, which was used in combination with a platinum agent and 5-FU in the EXTREME trial [1].

In our case report, adding cetuximab to carboplatin/5-FU led to a rapid reduction in the size of the facial mass, in facial oedema and in pain. Within 7 days of treatment, the patient's pain had reduced from 9/10 on a visual scale to 2/10 and after 3 cycles of treatment, pain was controlled without pain relief. Within 2 cycles of treatment, the facial mass had reduced to less than half its size prior to treatment initiation. The patient also reported an improvement in the ability to chew food. In addition, it was possible to administer cetuximab continuously as monotherapy for 7 months, during which time symptoms remained alleviated. It was eventually interrupted due to grade 2 mucositis and skin reactions. It is notable that facial pain returned on cetuximab cessation and decreased again following cetuximab resumption.

The response to the regimen of cetuximab and carboplatin/5-FU is particularly encouraging in view of the fact that it was achieved in a tumor that had less than six months earlier been irradiated and that had previously progressed rapidly on a combination of cisplatin and radiotherapy.

An association between platinum/5-FU chemotherapy, tumour response and symptom control in patients with recurrent and/or metastatic SCCHN was reported over ten years ago by Constenla et al.[9]. They reported that patients achieving an objective tumour response to treatment were more likely to obtain some symptom relief and improve their performance status than those not achieving a response. The phase III EXTREME trial showed that adding cetuximab to platinum-based chemotherapy significantly improved response rate compared with platinum-based chemotherapy alone [1]. A quality of life analysis of this trial reported that adding cetuximab to platinum/5-FU did not adversely affect global quality of life [10]. There were also some improvements with cetuximab/platinum-based therapy compared with platinum-based therapy alone in a number of parameters, including pain and swallowing [10].

The results of our case report are significant in that they demonstrate the speed with which symptom relief, associated with tumour mass reduction, can be achieved with cetuximab/carboplatin/5-FU for a patient with recurrent and/or metastatic SCCHN whose quality of life is severely compromised by their disease.

## Consent

Written informed consent was obtained from the patient for the publication of this case report and the accompanying images. A copy of the written consent is available for review by the Editor-in-Chief of this journal.

## Competing interests

RM has received reimbursement from Merck KGaA for participation in advisory boards and conferences. All other authors declare that they have no competing interests.

## Authors' contributions

RM has participated in the conception and design the manuscript, manuscript writing and provided the clinical case. RP has participated in the manuscript design and provided the clinical case. MC has participated in elaborating the CT scan figures and text. EV has participated in providing the clinical case and manuscript writting. SV has participated in the conception and design the manuscript and manuscript writing. Final approval of manuscript: Ricard Mesía, Ramon Palmero, Mònica Cos, Esther Vilajosana and Silvia Vázquez.

## References

[B1] Vermorken JB Mesia R Rivera F Remenar E Kawecki A Rottey S Erfan J Zabolotnyy D Kienzer HR Cupissol D Peyrade F Benasso M Vynnychenko I De Raucourt D Bokemeyer C Schueler A Amellal N Hitt R Platinum-based chemotherapy plus cetuximab in head and neck cancer N Engl J Med 2008 359 1116 1127 10.1056/NEJMoa0802656 18784101

[B2] Bonner JA Harari PM Giralt J Azarnia N Shin DM Cohen RB Jones CU Sur R Raben D Jassem J Ove R Kies MS Baselga J Youssoufian H Amellal N Rowinsky EK Ang KK Radiotherapy plus cetuximab for squamous-cell carcinoma of the head and neck N Engl J Med 2006 354 567 578 10.1056/NEJMoa053422 16467544

[B3] Bonner JA Harari PM Giralt J Cohen RB Jones CU Sur RK Raben D Baselga J Spencer SA Zhu J Youssoufian H Rowinsky EK Ang KK Radiotherapy plus cetuximab for locoregionally advanced head and neck cancer: 5-year survival data from a phase 3 randomised trial, and relation between cetuximab-induced rash and survival Lancet Oncol 2009 in press 19897418 10.1016/S1470-2045(09)70311-0

[B4] Forastiere A Koch W Trotti A Sidransky D Head and neck cancer N Engl J Med 2001 345 1890 1900 10.1056/NEJMra001375 11756581

[B5] Wong SJ Machtay M Li Y Locally recurrent, previously irradiated head and neck cancer: concurrent re-irradiation and chemotherapy, or chemotherapy alone? J Clin Oncol 2006 24 2653 2658 10.1200/JCO.2005.05.3850 16763279

[B6] Buccheri G Ferrigno D Tamburini M Karnofsky and ECOG performance status scoring in lung cancer: a prospective, longitudinal study of 536 patients from a single institution Eur J Cancer 1996 32A 1135 1141 10.1016/0959-8049(95)00664-8 8758243

[B7] Forastiere AA Metch B Schuller DE Ensley JF Hutchins LF Triozzi P Kish JA McClure S VonFeldt E Williamson SK Von Hoff DD Randomized comparison of cisplatin plus fluorouracil and carboplatin plus fluorouracil versus methotrexate in advanced squamous-cell carcinoma of the head and neck: a Southwest Oncology Group study J Clin Oncol 1992 10 1245 1251 1634913 10.1200/JCO.1992.10.8.1245

[B8] Gibson MK Li Y Murphy B Hussain MH DeConti RC Ensley J Forastiere AA Randomized phase III evaluation of cisplatin plus fluorouracil versus cisplatin plus paclitaxel in advanced head and neck cancer (E1395): an intergroup trial of the Eastern Cooperative Oncology Group J Clin Oncol 2005 23 3562 3567 10.1200/JCO.2005.01.057 15908667

[B9] Constenla DO Hill ME A'Hern RP Henk JM Rhys-Evans P Breach N Archer D Gore ME Chemotherapy for symptom control in recurrent squamous cell carcinoma of the head and neck Ann Oncol 1997 8 445 449 10.1023/A:1008203613364 9233523

[B10] Rivera Herrero F Hitt R Kawecki A Rottey S Peyrade F Vynnychenko I Curran D Kisker O Gross A Vermorken J Cetuximab plus platinum-based therapy firstline in patients with recurrent/metastatic (R/M) squamous cell carcinoma of the head and neck (SCCHN): a quality of life (QOL) analysis of the EXTREME trial Ann Oncol 2008 19 Suppl 8 Abstract 693PD

